# Nucleus accumbens deep brain stimulation in a rat model of binge eating

**DOI:** 10.1038/tp.2015.197

**Published:** 2015-12-15

**Authors:** W T Doucette, J Y Khokhar, A I Green

**Affiliations:** 1 Department of Psychiatry, Geisel School of Medicine at Dartmouth, Lebanon, NH, USA; 2 Department of Pharmacology and Toxicology, Geisel School of Medicine at Dartmouth, Lebanon, NH, USA; 3 The Dartmouth Clinical and Translational Science Institute, Dartmouth College, Lebanon, NH, USA

## Abstract

Binge eating (BE) is a difficult-to-treat behavior with high relapse rates, thus complicating several disorders including obesity. In this study, we tested the effects of high-frequency deep brain stimulation (DBS) in a rodent model of BE. We hypothesized that BE rats receiving high-frequency DBS in the nucleus accumbens (NAc) core would have reduced binge sizes compared with sham stimulation in both a ‘chronic BE' model as well as in a ‘relapse to chronic BE' model. Male Sprague–Dawley rats (*N*=18) were implanted with stimulating electrodes in bilateral NAc core, and they received either active stimulation (*N*=12) or sham stimulation (*N*=6) for the initial chronic BE experiments. After testing in the chronic BE state, rats did not engage in binge sessions for 1 month, and then resumed binge sessions (relapse to chronic BE) with active or sham stimulation (*N*=5–7 per group). A significant effect of intervention group was observed on binge size in the chronic BE state, but no significant difference between intervention groups was observed in the relapse to chronic BE experiments. This research, making use of both a chronic BE model as well as a relapse to chronic BE model, provides data supporting the hypothesis that DBS of the NAc core can decrease BE. Further research will be needed to learn how to increase the effect size and decrease deep brain stimulation-treatment outcome variability across the continuum of BE behavior.

## Introduction

The syndrome of binge eating (BE) is a component of a number of important disorders, including BE disorder, bulimia nervosa and obesity. With the lifetime incidence of BE increasing with obesity in the general population (affecting 30% of dieting obese individuals), BE contributes to significant morbidity, mortality and associated health-care costs.^1, 2^ As BE is very difficult to manage, the development of new treatments is of great public health importance.

Emerging evidence suggests that BE, like other appetitive disorders (for example, addictions), is associated with dysfunction of the brain reward circuit (BRC).^3, 4, 5, 6, 7^ This knowledge has led to both preclinical and clinical investigations using deep brain stimulation (DBS; inducing direct, focal modulation of the BRC) to treat disorders of appetitive behavior.^8, 9, 10^ DBS of the nucleus accumbens (NAc), a key element of the BRC, is currently under investigation as a potential therapy for major depression, eating disorders, substance use disorders and obesity.^11, 12, 13^ As DBS has already been established as a relatively safe and effective treatment for Parkinson's disease and other movement disorders, it would appear to be a potentially viable future treatment option for severe appetitive disorders.^14^

In rodents, a significant body of literature has implicated the NAc core and shell subregions in mediating reward cue-driven consumptive behaviors.^15, 16, 17^ DBS studies targeting the NAc to modify appetitive behaviors have had success within both subregions of the NAc, with some selectivity depending on the rewarding substance or the behavioral context being investigated.^4, 8, 9, 18^ One prior study of BE investigated *unilateral* DBS in the NAc *shell* and demonstrated a large decrease in binge size in *mice*.^19^ The present study was designed to test the hypothesis that *bilateral* NAc *core stimulation* in BE *rats* would lead to a reduction in binge size when performed during a chronic BE state as well as during relapse to chronic BE. The ultimate goal of this line of research is to move toward efficacious treatment of BE through neuromodulation of the BRC.

## Materials and methods

### Animals

Male Sprague–Dawley rats (*N*=24) were purchased from Harlan (South Easton, MA, USA) at 60 days of age and were individually housed on a reverse 12-h light/dark schedule with food and water *ad libitum*. ‘House chow' contained 28% protein, 58% carbohydrates and 18% fat by calories and 3.1 kcal g^−1^ (Harlan 2018S). Given a previously documented macronutrient preference for sugar and fat, a high-fat, high-sugar diet (‘sweet-fat diet'), which contained 19% protein, 36.2% carbohydrates and 44.8% fat by calories and 4.6 kcal g^−1^ (Teklad Diets 06415, South Easton, MA, USA), was used in this study to model BE in the rats. Following surgery, described below, 24 animals were randomly assigned into one of three intervention groups (*n*=8 animals per group) at the initiation of the study using a simple randomization. Group size was based on prior studies using DBS in rodent models of appetitive behavior.^19, 20^ Some animals had to be killed (*N*=6) over the course of the study for health reasons (for example, head-cap failure) and were removed from their respective groups for the analysis. As a result, the final group numbers for the intervention groups, described in more detail below, were as follows: ‘chronic BE state' (stim (*N*=12), sham (*N*=6)) and ‘relapse to BE' (sham → sham *(N*=6), stim → sham (*N*=5), stim → stim (*N*=7)). All experiments were carried out in accordance with the National Institute of Health Guide for the Care and Use of Laboratory Animals (NIH Publications No. 80-23) revised in 1996 and were approved by the Institutional Animal Care and Use Committee of Dartmouth College.

### Surgery

Following habituation to the animal facility (1 week), and before exposure to the sweet-fat diet, rats were implanted with bipolar electrodes. The animals were anesthetized with isoflurane inhalation (5% induction, 2% maintenance) and mounted in a stereotaxic frame. Custom bipolar electrodes (Plastics One, Roanoke, VA, USA) were implanted bilaterally into the NAc core, according to the following coordinates (relative to bregma: 1.2 mm anterior, 2.8 mm lateral and 7.6 mm ventral to the brain surface). Electrodes were implanted at a 4-degree angle to the perpendicular. Four stainless steel skull screws were placed around the implant sites. Mechanical etching using a razor blade was employed to increase the surface area of the rat skull, to which dental cement was applied (Dentsply, York, PA, USA). Animals were allowed to recover from the surgery for 1-week before the start of experiments.

### BE paradigm

#### Acquisition of the chronic BE state

The method used to induce BE in this study is a modification of previously published models, using a limited access protocol.^21, 22, 23^ Briefly, ‘sweet-fat diet' pellets were provided to the rats in addition to house chow and water within stimulation chambers daily for 2-h. Intake of the sweet-fat diet, regular chow and water was measured within the 2-h period. The previous 22-h consumption of house chow and water was also measured. Rats were tethered with external wires through a commutator to the stimulator before starting the binge sessions each day. This protocol was continued until a stable level of sweet-fat diet intake was obtained, at which point animals were considered to be in a *chronic BE state* (with <10% variation in sweet-fat diet intake over four consecutive sessions).

#### Relapse to the chronic BE state

Relapse, defined as the resumption of bingeing behavior during self-imposed or forced abstinence in humans, was modeled here in rats. Rats that had established a chronic BE state underwent 1 month of forced abstinence from BE without exposure to the cues previously associated with the binge sessions—including the sweet-fat pellets and the binge chambers. Relapse was assessed during the initial session in which the animals returned to the binge chambers and were given access to the sweet-fat pellets. Our model of ‘relapse' was adapted from previously published cue-induced relapse models that used conditioned stimulus cues to induce reinstatement of operant responding after extinction.^24, 25^ To more closely model relapse in clinical populations, however, we used a period of forced abstinence from both the unconditioned stimulus (consumption of the sweet-fat diet) and the associated conditioned stimuli (exposure to the binge chamber, tethering and olfactory cues associated with the food) without altering their association through extinction.

### Acute DBS

A current-controlled stimulator (S11, Grass Instruments, Quincy, MA, USA) with optical isolation units (PSIU6, Grass Technologies, Quincy, MA, USA) was used to generate a continuous train of monophasic pulses (60-μs pulse width at 150 Hz). The output of the stimulus isolator was monitored using a factory-calibrated oscilloscope (TPS2002C, Tektronix, Beaverton, OR, USA). The administered current output from this constant current stimulator was calibrated to 150 μA. During binge sessions in which animals received active stimulation, the stimulator was turned on immediately before animals had access to the sweet-fat pellet and turned off at the completion of the 2-h binge session. Rats were then disconnected from external wires and returned to home cages. The selection of parameters (pulse width, frequency and current intensity) was based on recent basic science and clinical work.^26, 27, 28, 29^

#### Intervention in the chronic BE state

Once animals reached the chronic BE state (described above), three 2-h binge sessions with intervention (‘intervention sessions') were run with each animal receiving either active or sham stimulation depending on their group assignment. The final number of animals per group after health exclusions for the sham group (no active stimulation) was 6 rats and for the active stimulation group was 12 rats. To assess for any residual effect of stimulation in subsequent binge sessions, all animals underwent three additional binge sessions without stimulation (‘post-intervention sessions') after the three intervention sessions. After the post-intervention sessions, animals remained in their home cage with *ad libitum* access to house chow and water for 1 month without access to the sweet-fat diet, exposure to the binge chamber or handling other than for cage changes.

#### Intervention in the relapse to the chronic BE state

**Figure 1:**
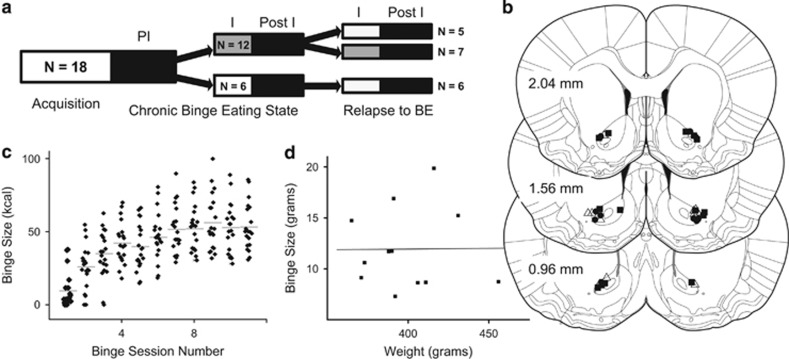
Experimental design and acquisition of the chronic binge-eating state. (**a**) Experimental design illustrating the behavioral states and corresponding progression of group sizes. White and black boxes denote sessions of no stimulation (sham), whereas gray boxes represent active stimulation. PI, pre-intervention; I, intervention; Post I, Post intervention. (**b**) Electrode locations displayed on representative coronal plates spanning the anterior–posterior dimension (A–P coordinates shown) using the following group symbol pairs: •, Stim→Stim; ▪, Stim→Sham; Δ, Sham→Sham. (**c**) Acquisition of bingeing behavior in all 18 animals showing the variation in binge size (kcal) across sessions. Binge sizes are displayed in kcal for comparison with the previously published model. Group averages in each session are shown with horizontal gray line. (**d**) Plot of binge size (g) versus body weight (g) in the chronic binge-eating state for all animals showing no significant relationship (*R*=0.0094, *P*=0.977).

When rats restarted binge sessions (in the relapse to the chronic BE state), they immediately entered three intervention sessions, followed by post-intervention sessions. The stimulation group from the previous chronic binge state experiments (*N*=12) was further divided, based on prior group assignment, into two subgroups that received either sham stimulation (stim → sham, *N*=5) or active DBS (stim → stim, *N*=7) during intervention sessions in the relapse to chronic BE state. The sham group from the chronic BE state experiment continued to receive sham stimulation (sham → sham, *N*=6) during intervention sessions in the relapse to chronic BE state (Figure 1a). These final group numbers reflect initial group assignment (*N*=8) minus the number of animals removed in each group during the course of the study because of health concerns.

### Verification of electrode placement

At the conclusion of the study, rats were injected with a lethal dose of sodium pentobarbital 200 mg kg^−1^ and perfused transcardially with normal saline (0.9% NaCl), and then with 4% paraformaldehyde fixative in 0.1 m phosphate-buffered saline. Electrodes then were removed. Whole brains were extracted from the crania, post-fixed for 24 h and then submerged in 20% sucrose in 0.1 m PBS for 48 h. Brains were frozen and cut by cryostat into 40-μm coronal sections, which then were mounted on glass slides and stained using cresyl violet.^30^ Electrode locations for all rats included in the study (*N*=18) are displayed in Figure 1b.

### Data analysis

Percent change in food intake from pre-intervention sessions in the chronic BE state ((grams consumed in the intervention session−average grams consumed in pre-intervention sessions)/average grams consumed in pre-intervention sessions × 100) was calculated for all sessions after a chronic BE state was achieved (<10% variation over four consecutive sessions). Intervention and post-intervention session data for both the chronic BE state and the relapse to chronic BE state were analyzed using two-way repeated-measures analysis of variance (RMANOVA), using time (session number) and intervention group (sham (*N*=6) versus stimulation (*N*=12)) as independent variables. When the analysis indicated that differences existed between intervention groups, *post hoc* pairwise comparisons between groups were made using the Bonferroni adjustment. When RMANOVA analysis indicated that significant differences existed, pairwise comparisons were tested at each session to help interpret significant group differences as well as group × time interactions from the RMANOVAs using a one-way ANOVA followed by *post hoc* analyses between groups on each day using the Tukey adjustment. Significance was determined at *P*<0.05. Data are expressed as mean (M)±s.e.m.

## Results

### Acquisition of BE

Implanted and tethered rats acquired binge behavior in ~8 days (Figure 1c), only a two to three session delay as compared with data from previously published unimplanted and tethered animals. As in the previously published model, animals in this study did not consume any of the house chow during the binge sessions despite its availability (data not shown). Water consumption was highly variable during the binge session and neither correlated with binge size nor did it significantly vary with interventions (data not shown). Binge sizes during the final three sessions shown in Figure 1c approximate the values observed when animals reached the chronic BE state (defined above) and illustrate the normal animal-to-animal variation. This inter-animal variation in binge size during a chronic BE state has been previously described by others with the outer quintiles defined as BE prone and BE resistant.^31, 32^ Interestingly, when binge sizes in the chronic BE state were plotted with the corresponding animal weights, there was no significant correlation (*R*=0.0094, *P*=0.977; Figure 1d). Therefore, we did not normalize binge size by animal weight in subsequent analyses.

### Chronic BE state

**Figure 2:**
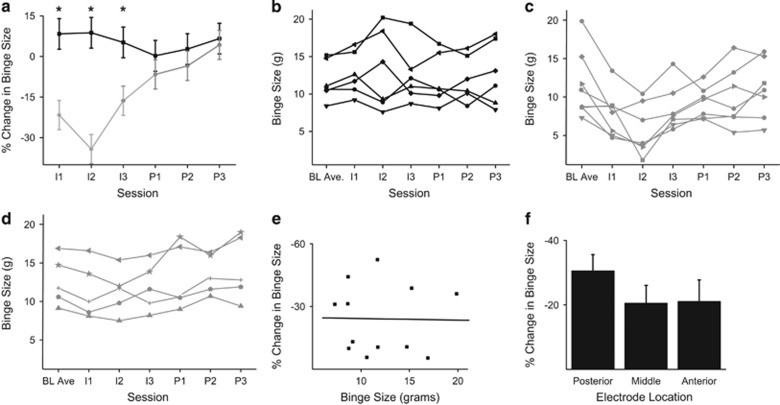
Chronic binge-eating state. (**a**) Binge size in DBS intervention group (gray circles) versus sham intervention group (black squares) across three intervention sessions (I1-3) and three post-intervention sessions (P1-3). Asterisks highlight sessions with significant differences between the sham and stimulation groups (*P*<0.05). (**b**) Individual rat binge sizes in the sham group across sessions shown in **a**. (**c**) Individual rat binge sizes (*N*=7) in the stimulation group that showed a reduction in binge size (>10% reduction in binge size on any intervention day) across sessions shown in **a**. (**d**) Individual rat binge sizes (*N*=5) in the stimulation group that failed to show a reduction in binge size across sessions shown in **a**. (**e**) Plot of pre-intervention binge size in the chronic binge-eating state (grams) versus average percent reduction in binge size in the stimulation group showing no significant relationship (*N*=12; *R*=−0.018, *P*=0.957). (**f**) Average percent reduction in binge size, binned by electrode location along the anterior–posterior axis (*N*=8 per group). Error bars in all panels: ±s.e.m.

The data shown in Figure 2a were analyzed using a repeated-measures ANOVA with a group (stimulation and sham stimulation) × session (three intervention and three post-intervention session) design. There was a main effect for group (F(1,17)=8.12, *P*=0.012), an interaction effect between group and session (F(1,16)=26.63, *P*<0.0001) and a main effect for session (F(1,16)=15.35, *P*=0.001). The primary finding (Figure 2a) was a reduction in binge size with bilateral NAc core DBS compared with sham stimulation. In addition, however, the data also highlighted the within-animal and between-animal variances across the intervention and post-intervention sessions (Figures 2b and c).

In order to determine which sessions were significantly different between groups, a subsequent session-by-session analysis of between-group differences using ANOVA was performed. This analysis showed a significant difference between the active stimulation group and the sham group on the 3 days of stimulation (I1 F(1,17)=13.033, *P*=0.002; I2 F(1,17)=11.232, *P*=0.004; I3 F(1,17)=10.253, *P*=0.006) but not on the post-stimulation days (P1 F(1,17)=0.83, *P*=0.376); P2 F(1,17)=0.65, *P*=0.43; P3 F(1,17)=0.06 *P*=0.81; Figure 2a). There was no significant difference in water consumption during the binge sessions between groups, consistent with prior work (data not shown). House chow consumption during the binge session was not included in the analysis because, as in the previously published model,^21^ it was not consumed during the binge sessions.

Although these data are promising, as noted above, the variability in treatment response observed in the active stimulation group both between-subjects and within-subjects from session to session was notable (See Figures 2b and d). In an effort to generate preliminary data to guide future explorations into the intersubject variation in treatment outcomes, we performed a pair of *post hoc* regression analyses to assess: (1) the relationship between the pre-intervention binge size in the chronic BE state and the average percent reduction in binge size (Figure 2e) and (2) the impact of electrode location within the NAc core along the anterior–posterior axis in relation to average percent reduction in binge size (Figure 2f). There was no correlation between the percent reduction in binge size with DBS and the pre-intervention binge size in the chronic BE state (*R*=−0.018, *P*=0.957). In addition, although a trend existed toward a larger reduction in binge size with electrode placement in the posterior third of the NAc core, there was no significant group effect on binge reduction with ANOVA (F(1,23)=4.11, *P*=0.056).

### Relapse to chronic BE state

**Figure 3:**
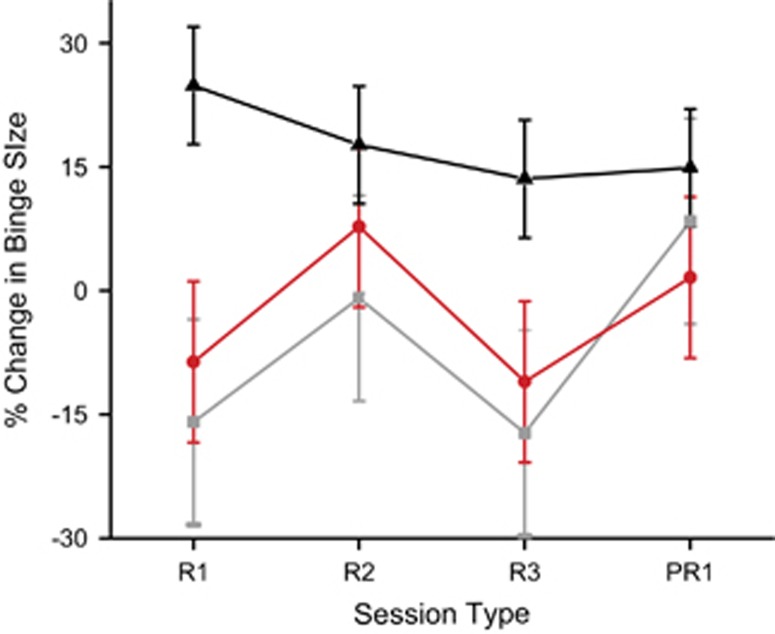
Relapse to the chronic binge-eating state. Percent change from the pre-intervention chronic binge-eating state for the three intervention groups: Stim →Stim (gray square), Stim→Sham (red circle), Sham→Sham (black triangle). R1-3 are intervention days and PR1 is the first post-intervention day with all groups getting sham stimulation. Error bars: ±s.e.m.

As shown in Figure 3, BE in animals administered DBS to the NAc core was similar to BE in sham animals assessed longitudinally within the relapse to chronic BE state. Unexpectedly, binge size did not appear to be significantly reduced by DBS in the ‘relapse' to chronic BE state as compared with the chronic BE state. The data shown in Figure 3 were analyzed using a RMANOVA with a group (stim→stim, stim→sham and sham→sham) × session (three intervention and one post-intervention session) design. No main effect of group (F(2,15)=2.97, *P*=0.082) was observed, with no interaction between groups and session (F(2,15)=1.17, *P*=0.34), or a main effect of session (F(1,15)=0.21, *P*=0.656). Therefore, despite the apparent increase in binge size from their previous baseline in the sham→sham group on the first day of relapse, a one-way ANOVA was not run to assess group differences during the first session, given the lack of significant effect of group in the RMANOVA.

## Discussion

The NAc is critical in mediating reward-seeking behaviors, including BE.^33^ DBS of this brain region has been shown to reduce consumption of cocaine, morphine, alcohol and high-fat chow.^19, 20, 26, 34^ In this study, we used a limited access model of BE in which rats were allowed 2 h to consume a highly palatable and calorically dense sweet-fat diet. As we hypothesized, NAc core DBS, administered during the 2-h limited access period, reduced binge size in the chronic BE state. However, NAc core DBS was not effective in the relapse to the chronic BE state with no significant difference seen between groups.

Taken together, the work presented here and by Halpern *et al.*^19^ suggests that stimulation of the NAc produces a significant reduction in binge size in a chronic BE model despite differences in animal species (mouse versus rat), subregion of the NAc targeted (unilateral shell versus bilateral core) and the type of palatable food (high-fat versus sweet-fat). Moreover, both studies show that binge reduction does not continue beyond the sessions in which the animals receive stimulation. Interestingly, both studies also highlight that when behavioral context changes from a chronic BE state to alternative behaviors of palatable food consumption (that is, diet-induced obesity or a binge relapse model), the impact of NAc DBS appears to be less robust.

In the diet-induced obesity model used in the Halpern study, the decreased effectiveness of NAc DBS to reduce palatable food consumption over time may be related to continuous stimulation, which has been shown to induce differential network modulation compared with acute stimulation. The evolving DBS-induced network changes demonstrated by Ewing and Grace^35^ may explain why in some circumstances the treatment outcomes diminish with continuous stimulation, whereas others, like improvement of depression symptoms with subgenual cingulate DBS, take time to develop. Future work is needed to determine how continuous stimulation of the NAc core compares with stimulation in the NAc shell to reduce weight in a diet-induced obesity model.

In the relapse to chronic BE state, the decreased efficacy of NAc stimulation may stem from an increased drive/motivation to consume the palatable food. This is consistent with prior studies using a cue-induced model of relapse to substances of abuse that have shown an ‘incubation of craving' with prolonged abstinence driving enhanced responding for drug when animals are re-exposed to previously associated cues.^24, 36, 37^ Although cue-induced craving was not assessed here, it is possible that a similar underlying mechanism contributed to the decreased efficacy of DBS to reduce binge size during these sessions. To achieve the eventual goal of using focal neural modulation to simultaneously treat a spectrum of dysfunctional food consumption (BE, overeating and obesity), and to prevent relapse to these behaviors, further characterization using other behavioral paradigms will be needed to improve treatment outcomes.

Whereas most studies employing NAc DBS in rats to reduce appetitive behaviors have focused on the shell subregion,^38, 39, 40, 41^ some studies such as ours have assessed the effects of high-frequency DBS of the NAc core on consumptive behaviors. Alcohol drinking in rats was significantly reduced when animals received DBS of the NAc core,^20, 41^ and chronic high-frequency stimulation of the NAc core significantly reduced conditioned place preference for morphine.^34^ Interestingly, some studies have failed to show NAc subregion specificity of treatment outcomes, as demonstrated by Hamilton *et al.* in their investigation of cocaine-induced reinstatement of cocaine self-administration.^42^ Taken together, this body of work suggests that modulation of the NAc (core as well as shell) using DBS or another non-invasive stimulation method should continue to be investigated for the treatment of appetitive behaviors.

A number of potential limitations should be noted. In terms of our primary outcome, one main concern stems from the type of stimulation that we used (monophasic). This type of waveform is no longer used clinically as it can be more damaging (with chronic stimulation) than biphasic stimulation. For this study, limiting the stimulation period to the binge session appeared to prevent significant unintentional lesioning of adjacent tissue, with no significant lesions observed during histologic examination. However, we cannot rule out some degree of local damage as a confounding factor in the outcome of this study. A second concern relates to the fact that the group sizes were not equal at the conclusion of the study because of unbalanced exclusions between treatment groups because of animal health over the duration of the study. Third, a fourth arm of the study (sham → stim) would have been useful in addressing the effects of stimulation in the relapse experiments if a significant effect had been identified. However, as the effect of stimulation in the chronic BE state did not persist beyond the treatment sessions, the contribution of stimulation that occurred more than 30 days before the relapse sessions was probably minimal. Prior work has also demonstrated no long-term influence of NAc DBS on locomotor activity, suggesting that our results were probably not because of motor effects of the DBS.^43^

Data from this study highlight an important limitation still impeding more widespread use of focal neural modulation: the variable and even unpredictable treatment outcomes. Preliminary evaluation of potential sources that could contribute to variance in DBS outcomes for BE revealed no significant correlations within the cohort of animals investigated. The variance in DBS outcomes could not be accounted for by either inter-animal variance in baseline binge size or electrode placement within the NAc core along the anterior–posterior axis. However, as this study was not explicitly designed to identify and test sources that could contribute to variance in DBS treatment outcomes for BE, and, moreover, *post hoc* analyses did reveal a trend toward significance, further studies will be needed to directly investigate whether variations in electrode placement within the NAc could underlie the observed DBS outcome variance.

We acknowledge that interindividual heterogeneity in binge size, known to exist in the Sprague–Dawley rat population, could theoretically underlie the observed variation in DBS outcomes in the chronic BE state. This behavioral heterogeneity has been well documented in the literature, where the outer quintiles of binge size variation have been defined as BE prone (upper quintile—BE prone) and BE resistant (lowest quintile—BE resistant).^31, 32^ Interestingly, however, differences in the pre-intervention binge size of animals in the chronic BE state did not correlate with the percent reduction in binge size induced by DBS. Therefore, future studies will be needed to better understand how variations in baseline binge size, electrode placement, stimulation parameters or natural variation in reward circuit function may relate to variations in DBS treatment outcomes to reduce BE.

As in this animal model of BE, the hope for advancing focal neural modulation lies within potential personalization, such that an individual's unique brain circuit dysfunction being targeted by DBS could be used as an intermediate marker to guide optimization of electrode placement and selection of stimulation variables. While a significant body of work has helped identify relevant circuits, more work is needed to characterize the relevant features of BRC activity that drive dysfunctional appetitive behavior. Further studies that capture circuit activity during behaviors of interest while stimulation parameters are manipulated and stimulation targets are varied will yield valuable information. Until these domains can be captured with adequate detail (behavior, circuit function and their modulation by variations in electrode placement and stimulation parameters), our ability to manipulate neural circuits in behaviorally meaningful ways will likely remain rudimentary. A more complete delineation of the inter-relationship of these domains could help guide the design of a closed loop system that could concurrently modulate stimulation parameters, adapting to each individual's circuit activity and its changes over time in order to allow for more nuanced and individually tuned focal neural modulation.

## Conclusion

In this study, use of DBS in a rat model that combines a chronic BE state with a relapse to BE provides evidence that BE behavior can be manipulated by acute DBS within the NAc core. The results provide additional preclinical support for the potential utilization of focal neural modulation of the BRC to reduce binge behavior in patients.

## Notes

By way of disclosure, in the past 3 years, Dr Green has received research grants from Novartis, Janssen and Alkermes to support research studies, and has served as an uncompensated consultant to Otsuka and Alkermes and as an (uncompensated) member of a data safety monitoring group for Eli Lilly. The remaining authors declare no conflicts of interest.
